# Network-based *in silico* drug efficacy screening

**DOI:** 10.1038/ncomms10331

**Published:** 2016-02-01

**Authors:** Emre Guney, Jörg Menche, Marc Vidal, Albert-László Barábasi

**Affiliations:** 1 Center for Complex Networks Research (CCNR) and Department of Physics, Northeastern University, 177 Huntington Avenue, 11th floor, Boston, Massachusetts 02115, USA; 2 Center for Cancer Systems Biology (CCSB) and Department of Cancer Biology, Dana-Farber Cancer Institute, 450 Brookline Avenue, Boston, Massachusetts 02215, USA; 3 Center for Network Science, Central European University, Nador utca 9, 1051 Budapest, Hungary; 4 Department of Genetics, Harvard Medical School, 77 Avenue Louis Pasteur, Boston, Massachusetts 02115, USA; 5 Department of Medicine, Brigham and Women's Hospital, Harvard Medical School, 75 Francis Street, Boston, Massachusetts 02115, USA

## Abstract

The increasing cost of drug development together with a significant drop in the number of new drug approvals raises the need for innovative approaches for target identification and efficacy prediction. Here, we take advantage of our increasing understanding of the network-based origins of diseases to introduce a drug-disease proximity measure that quantifies the interplay between drugs targets and diseases. By correcting for the known biases of the interactome, proximity helps us uncover the therapeutic effect of drugs, as well as to distinguish palliative from effective treatments. Our analysis of 238 drugs used in 78 diseases indicates that the therapeutic effect of drugs is localized in a small network neighborhood of the disease genes and highlights efficacy issues for drugs used in Parkinson and several inflammatory disorders. Finally, network-based proximity allows us to predict novel drug-disease associations that offer unprecedented opportunities for drug repurposing and the detection of adverse effects.

## Abstract (web summary)

Attempts to predict novel use for existing drugs rarely consider information on the impact on the genes perturbed in a given disease. Here, the authors present a novel network-based drug-disease proximity measure that provides insight on gene specific therapeutic effect of drugs and may facilitate drug repurposing.

## Graphical abstract

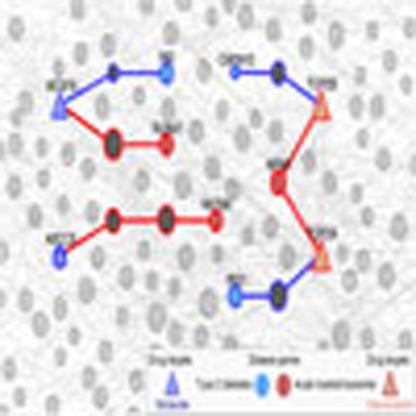

The emergence of most diseases cannot be explained by single-gene defects, but involve the breakdown of the coordinated function of distinct gene groups^1^. Consequently, to be successful, drug development must shift its focus from individual genes that carry disease-associated mutations towards a network-based perspective of disease mechanisms. We continue to lack, however, a network-based formalism to explore the impact of drugs on proteins known to be perturbed in a disease.

Network-based approaches have already offered important insights into the relationship between drugs and diseases. For example, the analysis of targets of US Food and Drug Administration (FDA) approved drugs and disease-related genes in Online Mendelian Inheritance in Man (OMIM)^2^ revealed that most drug targets are not closer to the disease genes in the protein interaction network than a randomly selected group of proteins^3^. This suggests that traditional drugs lack selectivity towards the genetic cause of the disease, targeting instead the symptoms of the disease. At the same time, several network-based approaches have focused on predicting novel targets and new uses for existing drugs^4^,^5^. Current approaches rely on target profile similarity, defined by either the number of targets two drugs share^6^,^7^ or the shortest paths between the drug targets in the interactome^8^,^9^,^10^,^11^. However, the existing literature-derived interaction sets are incomplete^12^ and biased towards more studied proteins, like drug targets and disease proteins^13^, shortcomings ignored by the existing network-based methods. In this study, we introduce an unsupervised and unbiased network-based framework to analyse the relationships between drugs and diseases. Recent studies have demonstrated that the genes associated with a disease tend to cluster in the same network neighborhood, called the disease module, representing a connected subnetwork within the interactome rich in disease proteins^12^,^14^. We, therefore, hypothesized that for a drug to be effective for a disease, it must target proteins within or in the immediate vicinity of the corresponding disease module. To test this hypothesis, we integrate protein–protein interaction, drug-disease association and drug-target association data allowing us to analyse the topological characteristics of drug targets with respect to the disease proteins. We propose a drug-disease proximity measure that helps us quantify the therapeutic effect of drugs, distinguishing non-causative and palliative from causative and effective treatments and offering an unsupervised approach to uncover novel uses for existing drugs.

## Results

### Proximity between drugs and diseases in the interactome

We start with all 1,489 diseases defined by Medical Subject Headings (MeSH) compiled in a recent study^12^ (Methods section). For each disease, we retrieve associated genes from the OMIM database^2^ and the GWAS catalog^15^. We focus on the diseases with at least 20 disease-associated genes in the human interactome such that the diseases are genetically well characterized and are likely to induce a module in the interactome^12^. We gather the drug-target information on FDA approved drugs from DrugBank^16^ and the indication information (the diseases the drug is used for) from the medication-indication resource high-precision subset (MEDI-HPS)^17^, which is then filtered by strong literature evidence using Metab2MeSH (ref. 18) to represent a high-confidence drug-disease association data set. In total, we identify 238 drugs whose indication matches 78 diseases and whose targets are in the human interactome containing 141,150 interactions between 13,329 proteins. Several of these drugs are recommended for more than one disease, resulting in 402 drug-disease associations between 238 drugs and 78 diseases. The average number of targets in the network per drug is *n*_target_=3.5 and the mean degree of the targets is *k*_target_=28.6, larger than the interactome's average degree *k*=21.2 (Supplementary Fig. 1), a difference that we attribute to the literature bias towards drug targets.

**Figure 1:**
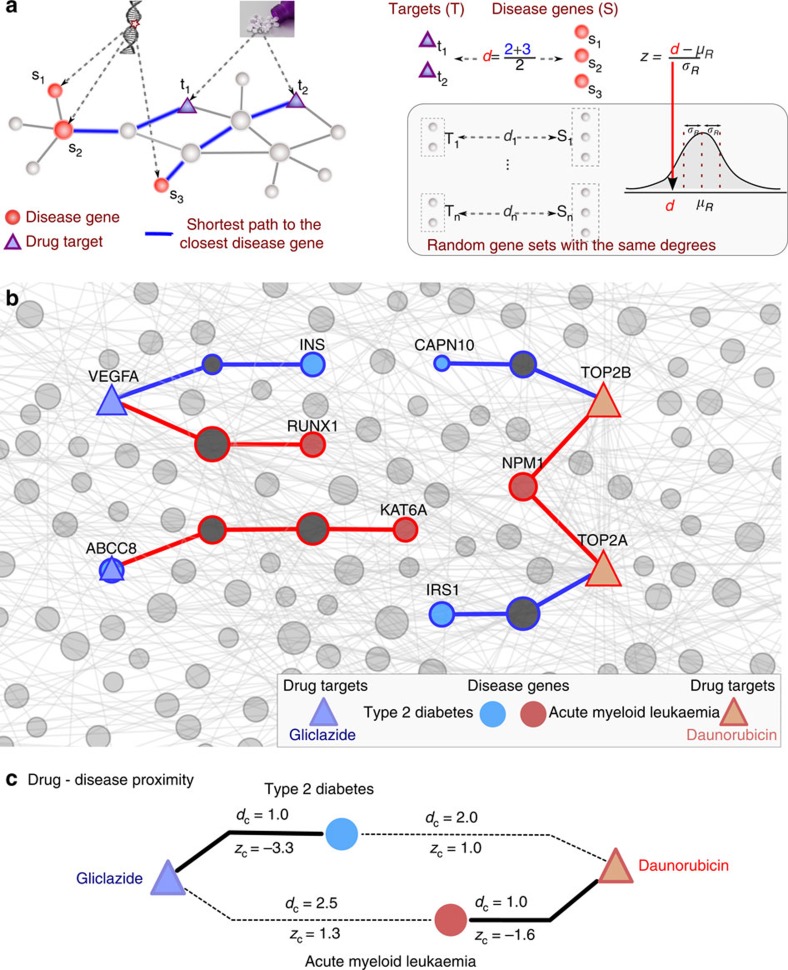
Network-based drug-disease proximity. (**a**) Illustration of the closest distance (*d*_c_) of a drug *T* with targets *t*_1_ and *t*_2_ to the proteins *s*_1_, *s*_2_ and *s*_3_ associated with disease *S*. To measure the relative proximity (*z*_c_), we compare the distance *d*_c_ between *T* and *S* to a reference distribution of distances observed if the drug targets and disease proteins are randomly chosen from the interactome. The obtained proximity *z*_c_ quantifies whether a particular *d*_c_ is smaller than expected by chance. To account for the heterogeneous degree distribution of the interactome and differences in the number of drug targets and disease proteins, we preserve the number and degrees of the randomized targets and disease proteins. (**b**) The shortest paths between drug targets and disease proteins for two known drug-disease associations: Gliclazide, a T2D drug with two targets and daunorubicin, a drug used for AML that also has two targets in the interactome. The subnetwork shows the shortest paths connecting each drug target to the nearest disease proteins. Proteins are coloured with respect to the disease they are associated with: T2D (blue) and AML (red). Drug targets are represented as triangles and coloured according to whether they are targets of gliclazide (light blue) and daunorubicin (brown). Blue and red links illustrate the shortest path from the drug targets to the nearest disease proteins (of T2D and AML, respectively). Node size scales with the degree of the node within the subnetwork. In case of multiple disease proteins with the equal shortest path lengths to the target, the disease protein with lowest degree in the interactome is shown. (**c**) The proximity *z*_c_ of gliclazide and daunorubicin to T2D and AML, indicating low *z*_c_ for the recommended use of these drugs and high *z*_c_ for their non-recomended use.

To investigate the relationship between drug targets and disease proteins, we develop a relative proximity measure that quantifies the network-based relationship between drugs and disease proteins (proteins encoded by genes associated with the disease). For this, for each drug-disease pair, we compare the network-based distance *d* between the known drug targets and the disease proteins to the expected distances *d*_rand_ between them if the target-disease protein sets are chosen at random within the interactome (Methods section). We initially focus on two distance measures *d* to determine the relative proximity: (i) The most straightforward measure is the average shortest path length, *d*_s_, between all targets of a drug and the proteins involved in the same disease; (ii) Acknowledging that a drug may not necessarily target all disease proteins, we also use closest measure, *d*_c_, representing the average shortest path length between the drug's targets and the nearest disease protein. In this case, we have *d*_c_=0 only if all drug targets are also disease proteins. For both distance measures, *d*_s_ and *d*_c_, the corresponding relative proximity *z*_s_ and *z*_c_ captures the statistical significance (*z*-score, 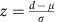) of the observed target-disease protein distance compared with the respective random expectation. Figure 1a illustrates the calculation of the relative proximity *z*_c_ using the closest measure *d*_c_, which, as we show later, outperforms other distance measures.

To demonstrate the utility of the relative proximity, Fig. 1b shows the shortest paths between drug targets and disease proteins for two known drug-disease associations: Gliclazide–type 2 diabetes (T2D) and daunorubicin–acute myeloid leukaemia (AML). Gliclazide binds to ATP-binding cassette sub-family C member 8 (ABCC8) and vascular endothelial growth factor A and stimulates pancreatic beta-islet cells to release insulin. *ABCC8* is a known T2D gene (MIM:600509) and there is at least one protein associated with T2D within two steps of vascular endothelial growth factor A's neighborhood corresponding to an average distance of *d*_c_=1.0 between the drug and the disease using the closest measure. The relative proximity between the drug and the disease is *z*_c_=−3.3, suggesting that the targets of gliclazide are closer to the T2D proteins than expected by chance (Fig. 1c). Similarly, the relative proximity of daunorubicin, an anthracycline aminoglycoside inhibiting the DNA topoisomerase II (*TOP2A* and *TOP2B*), to AML is *z*_c_=−1.6, offering network-based support for daunorubicin's therapeutic effect in AML. As a negative control, we measure the relative proximity of gliclazide to AML and daunorubicin to T2D, pairings whose efficacies are not known. In both cases, the disease proteins and drug targets are not closer than expected for randomly selected protein sets (*z*_c_=1.3 and *z*_c_=1.0, respectively), suggesting that these drugs do not target the disease module of other diseases, but they are specific to the module of the disease they are recommended for.

To generalize these findings, we group all possible 18,564 drug-disease associations between 238 drugs and 78 diseases into 402 known (validated) drug-disease associations that are reported in the literature (like gliclazide and T2D) and the remaining 18,162 unknown drug-disease associations that are not known (and are unlikely) to be effective (Supplementary Data 1). For example, we do not expect gliclazide to be more effective on AML than any other randomly chosen drug. Yet, a few of the 18,162 unknown drug-disease pairs may correspond to effective treatments, representing novel candidates for drug repurposing, challenging us to identify which ones. Consistent with previous observations^3^, only in 62 of the 402 known drug-disease associations (15.4%), a drug-target coincides with a disease protein. On the other hand, in 490 of 18,162 unknown drug-disease pairs (2.7%) the drug targets are known disease proteins, but not associated with the drug's actual disease indication. Although in both classes (known and unknown), the overlap between drug targets and disease proteins is low, the much higher ratio among known drug-disease associations (Fisher's exact test, odds ratio=6.6, two-sided *P*=5.2 × 10^−27^) suggests that direct targeting of known disease proteins is a rare but important therapeutic component in disease treatment.

### Drugs target the local neighborhood of the disease proteins

**Figure 2:**
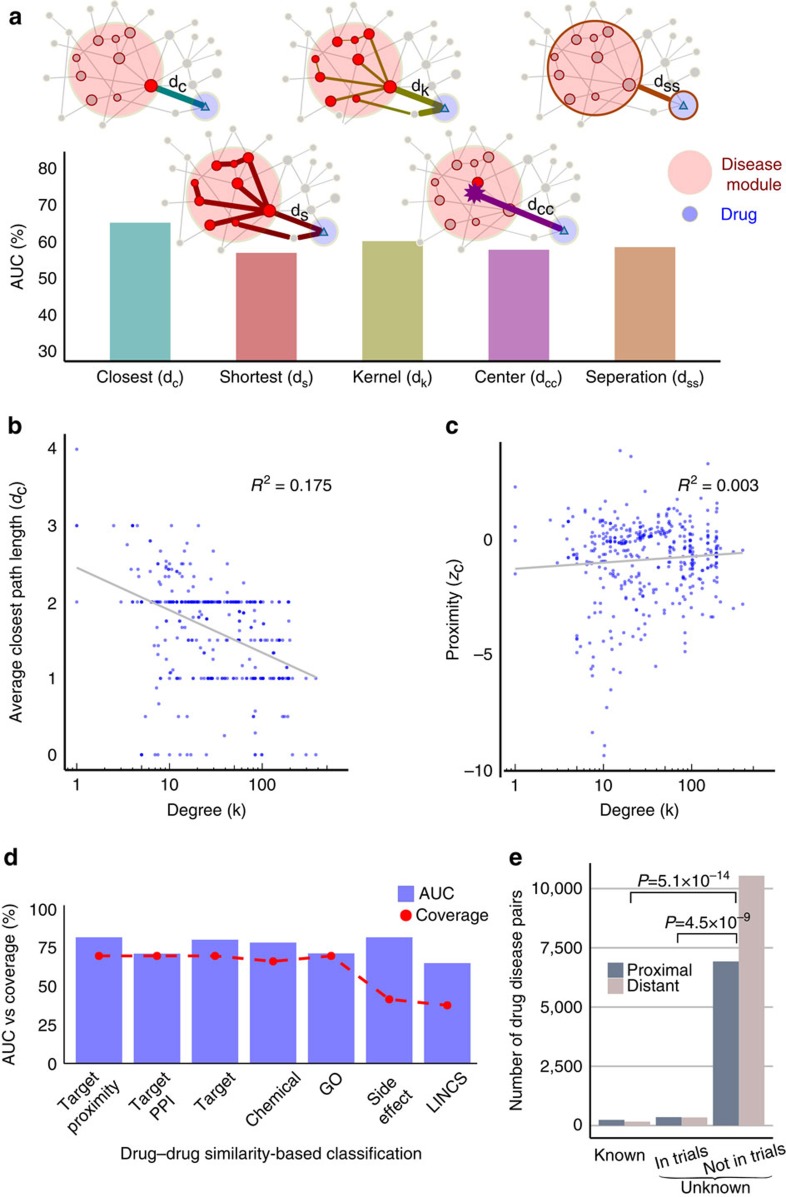
Validating drug-disease proximity. (**a**) AUC is shown for relative proximity, *z*, calculated using five different distance measures. The closest measure, *d*_c_, considers the shortest path length from each target to the closest disease protein, the shortest measure, *d*_s_ averages over all shortest path lengths to the disease proteins. See the text for the definition of the kernel (*d*_k_), centre (*d*_cc_) and separation (*d*_ss_) measures. (**b**) Average shortest path length between drug targets and disease proteins versus average drug-target degree for known drug-disease pairs. (**c**) Drug-disease proximity versus average degree of drug targets for known drug-disease pairs. (**d**) The plot shows AUC and coverage values for drug similarity-based measures based on the relative proximity between the targets (target proximity), the interactome-based distance between the targets (target PPI), sharing drug targets (target), chemical similarity (chemical), GO terms shared among the targets (GO), common differentially regulated genes in the perturbation profiles of the two drugs in LINCS database (LINCS), and common side effects (side effect). Coverage is defined as the percentage of drug-disease associations for which the method can make predictions. (**e**) Number of proximal and distant drug-disease pairs among known and unknown drug-disease associations (Fisher's exact test, odds ratio=2.1 and *P*=5.1 × 10^−14^). The unknown drug-disease associations are further categorized based on whether the association is in clinical trials (in trials) or not (not in trials, Fisher's exact test, odds ratio=1.6, *P*=4.5 × 10^−9^).

We first test how well relative proximity discriminates the 402 known drug-disease pairs from the 18,162 unknown drug-disease pairs by comparing the area under Receiver Operating Characteristic (ROC) curve (AUC, Methods section) for different distance measures. In addition to the closest (*d*_c_) and shortest (*d*_s_) measures discussed above, we measure relative proximity between a drug and a disease using three other network-based distance measures: (i) the kernel measure, *d*_k_, which downweights longer paths using an exponential penalty, (ii) the centre measure, *d*_cc_, which is the shortest path length between the drug targets and the disease protein with the largest closeness centrality among the disease proteins, (iii) the separation measure, *d*_ss_, that records the sum of the average distance between drug targets and disease proteins using the closest measure and subtracts it from the average shortest distance between drug targets and disease proteins. We find that the relative proximity defined by the closest measure *d*_c_ (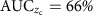) offers the best discrimination among the known and unknown drug-disease pairs (Fig. 2a), outperforming the shortest (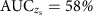, DeLong's AUC difference test *P*=5.1 × 10^−7^), the kernel (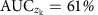, *P*=4.7 × 10^−4^), the centre (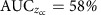, *P*=1.2 × 10^−5^), and the separation (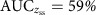, *P*=2.1 × 10^−4^) measures.

The superior performance of the closest measure suggests that drug targets do not have to be close to all proteins implicated in a disease. That is, drugs tend to affect a subset of the disease module rather than targeting the disease module as a whole. Indeed, we find that most drugs exert their therapeutic effect on disease proteins that are at most two links away (Supplementary Fig. 2 and Supplementary Note 1). Note also that relative proximity corrects for the biases of the traditional shortest path-based measures: the closest distance is significantly anti-correlated with the number of interactions the target proteins have (Spearman's rank correlation coefficient *ρ*=−0.46, *P*=8.6 × 10^−23^), whereas relative proximity associated with the closest distance show no correlation with degree (*ρ*=−0.01, *P*=0.84, Fig. 2b,c, Supplementary Fig. 3 and Supplementary Note 2).

### Proximity improves on existing drug repurposing approaches

The increasing interest in reusing existing drugs for novel therapies has recently given rise to various approaches that aim to identify candidate drugs with similar characteristics to known drugs used in a disease^7^,^8^,^19^,^20^. We use interactome-based drug-disease proximity to define similarity between two drugs and compare it with existing approaches defining similarity through (i) the shortest path distance between their targets in the interactome, (ii) common targets, (iii) chemical similarity, (iv) Gene Ontology (GO) terms shared among their targets, (v) common differentially regulated genes in the perturbation profiles of the two drugs in Library of Integrated Network-based Cellular Signatures (LINCS) database (lincsproject.org) and (vi) common side effects given in Side Effect Resource (SIDER) (ref. 21) (Supplementary Note 3). We find that proximity-based similarity discriminates known drug-disease pairs from unknown drug-disease pairs better than most of the existing similarity-based methods (AUC_targetproximity_=81%, Fig. 2d). The increase in the AUC is significant compared with using shortest path-based similarity (AUC_targetPPI_=71%, *P*=7.4 × 10^−14^), chemical similarity (AUC_chemical_=78%, *P*=0.03), functional similarity (AUC_GO_=71%, *P*=4.8 × 10^−18^) and expression profile similarity (AUC_LINCS_=65%, *P*=2.8 × 10^−20^). Proximity-based similarity definition outperforms the similarity definition based on shared targets, yet the improvement is not significant (AUC_target_=80%, *P*=0.12). Despite having comparable accuracy (AUC_sideeffect_=81%, *P*=0.56), the side effect similarity-based method is only applicable to less than half of the drug-disease pairs.

Although similarity-based methods are powerful in discriminating known drug-disease pairs from unknown drug-disease pairs, they have two main drawbacks: (i) these methods rely on the existing knowledge of drug and disease information, making them prone to overfitting and (ii) they fail to provide insights on the drug mechanism of action. Gene expression profile consistency based approaches aim to overcome these limitations by investigating correlations between the expression signatures of drug perturbations and the expression profiles in diseases^22^,^23^. We use the drug and disease signatures in drug versus disease (DvD) resource^24^ and calculate a Kolomgorov-Smirnov statistic-based enrichment score for the 1,980 (95 known, 1,885 unknown) drug-disease pairs that are in the DvD data set. We show that, proximity yields better accuracy than expression correlation-based prediction of drug-disease associations (AUC_proximity_=63% versus AUC_DvD_=53%, *P*=0.01, Supplementary Note 4). Though, the poor performance of the expression based approach is surprising, it is consistent with a recent systematic analysis reporting similar AUC values^25^. Therefore, proximity provides an alternative to the drug similarity and gene expression based repurposing approaches that can offer an interactome-based explanation towards the drug's effect on a disease. Their combination, though, could offer increased predictive power, given the orthogonal nature of the information the two classes of methods use.

### Proximity is a good proxy of therapeutic effect

**Figure 3:**
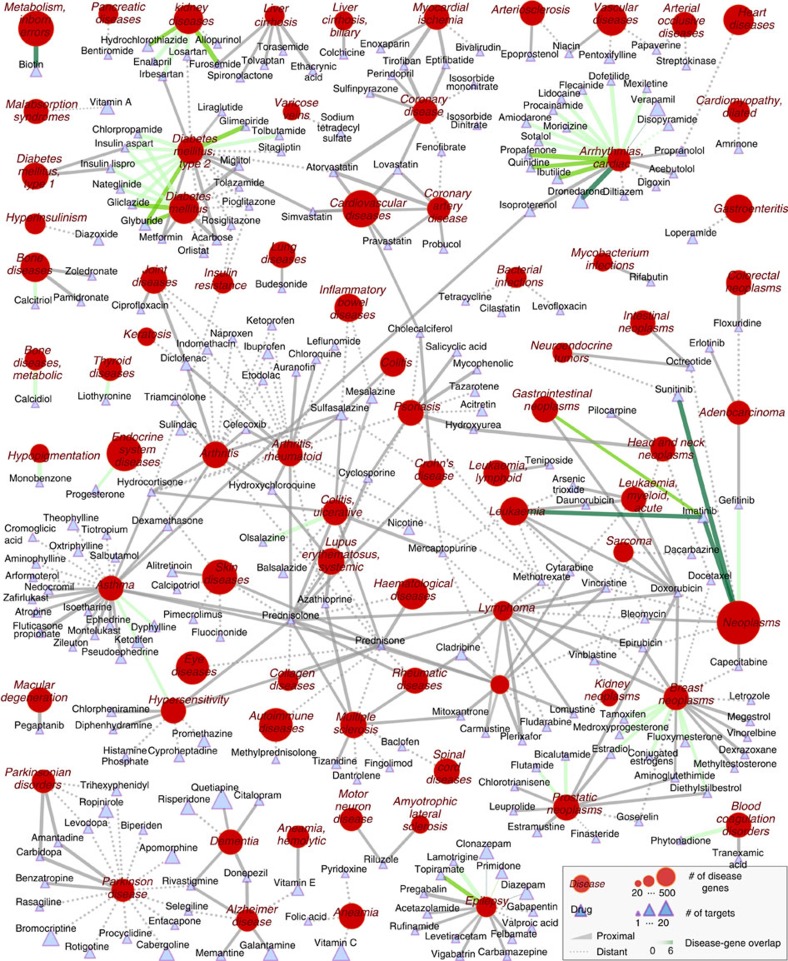
Known drug-disease associations. For each known drug-disease association, we connect the drug to the disease it is used for, the link style indicating whether the drug is proximal (solid) or distant (dashed) to the disease. The line colour represents the number of overlapping proteins between drug targets and disease proteins (0, grey; 6, dark green). Node shape distinguishes drugs (triangles) from diseases (circles). The node size scales with the number of proteins associated with the disease and with the number of targets of the drug.

The effectiveness of proximity as an unbiased measure of drug-disease relatedness prompts us to ask: Are drugs (drug targets) that are closer to the disease (disease proteins) more effective than distant drugs? To answer this, we define a drug to be proximal to a disease if its proximity follows *z*_c_≤−0.15, and distant otherwise (Methods section). This threshold is chosen as it offers good coverage of known drug-disease associations and few false positives (Supplementary Fig. 4 and Supplementary Note 5), helping us arrive to several key findings:

1. Known drugs are more proximal to their disease: For 237 of the 402 known drug-disease associations (59%), the drugs are proximal to the disease they are indicated for (Fig. 2e). At the same time, drugs are proximal in 7,276 of the 18,162 unknown drug-disease associations (40%), representing numerous potential candidates for drug repurposing. The ratio of known drug-disease associations among proximal drug-disease associations compared with the same ratio among distant drug-disease associations is statistically highly significant (Fisher's exact test, odds ratio=2.1, *P*=5.1 × 10^−14^). In other words, a drug whose targets are proximal to a disease is twice more likely to be effective for that disease than a distant drug.
2. Proximal drugs are more likely to be tested in clinical trials: The proximal but currently unknown drug-disease pairs are significantly over-represented in clinical trials compared with the distant unknown drug-disease pairs (353 proximal versus 341 distant drug-disease pairs, odds ratio=1.6, *P*=4.5 × 10^−9^).
3. Most known drugs are not exclusive: We examine the enrichment of known drug-disease associations among significantly proximal (that is, *z*_c_≤−2) drug-disease pairs and observe a significant increase in the ratio of known drug-disease pairs compared with unknown pairs (odds ratio=5.2, *P*=2.6 × 10^−27^). However, only 79 out of 402 known drug-disease pairs are significantly proximal to each other. Therefore, a drug should be sufficiently selective (that is, proximal to the disease) to have therapeutic effect but not necessarily exclusive (significantly proximal to the disease).
4. Proximity can highlight non-trivial associations: We find that in 18 known drug-disease pairs in which all the drug targets are also disease proteins, the drugs are proximal to the disease as one would expect. On the other hand, in 44 pairs for which at least one but not all of the drug targets are disease proteins, all the drugs are proximal to the disease with the only exception of disopyramide, a cardiac arrhythmia drug (Fig. 3). In 176 of the remaining 340 known drug-disease associations for which the drug targets do not coincide with any of the disease proteins, the drug targets are proximal to the disease, indicating that the interactome can highlight non-obvious drug-disease associations in which the drug does not directly target known disease proteins.

### Pinpointing palliative treatments using proximity

**Figure 4:**
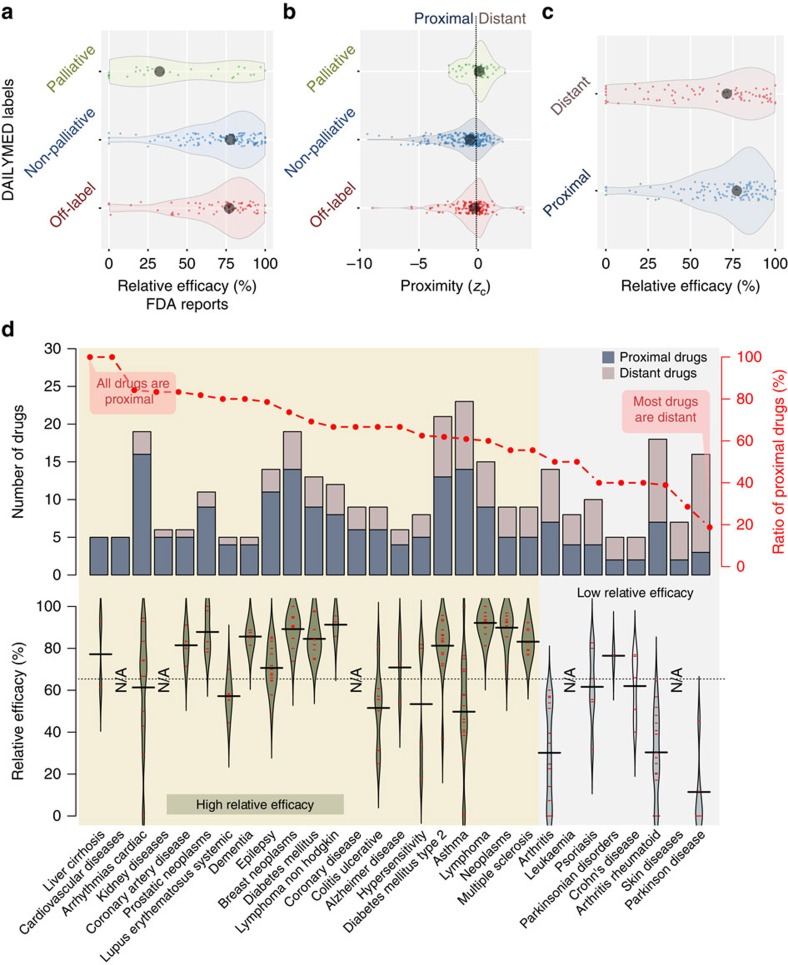
Drug-disease proximity and efficacy. (**a**) The distribution of RE scores calculated using FDA Adverse Event Reporting System for palliative (*n*=50), non-palliative (*n*=219) and off-label (*n*=133) drug-disease pairs annotated based on DailyMed description. A drug-disease pair is marked palliative if the indication in DailyMed referred to the non-causative use of the drug in that disease and non-palliative otherwise. If the indication is not in the label, then it is marked as off-label. The median within each group is shown as a black dot. The contours represent the probability density of the data points based on kernel density. Palliative uses have lower RE scores compared with non-palliative (one-sided Mann–Whitney *U* test =7.3 × 10^−5^) and off-label uses (*P*=7.6 × 10^−4^). (**b**) The distribution of drug-disease proximity for palliative, non-palliative and off-label drug-disease pairs. The palliative uses have higher proximity values (*P*=4.0 × 10^−5^ and *P*=0.02 compared with non-palliative and off-label uses, respectively). (**c**) The distribution of RE for proximal (*n*=237) versus distant (*n*=165) drug-disease pairs. The proximal drug-disease pairs have higher RE scores (*P*=0.04). (**d**) (Top panel) For each disease, the number of known drugs that are proximal to the disease (dark blue) compared with the number of distant drugs (light brown). The ratio of proximal drugs to all drugs is shown in red. The plot is split into two regions horizontally based on the ratio of proximal drugs: the diseases for which (i) more than half of the drugs are proximal (yellow background) and (ii) the rest (grey background). (Bottom panel) the RE scores of drugs for each disease are shown as red lines and the curve corresponds to the probability density estimate. The median within each disease is drawn by a solid line, whereas the median RE over all the diseases is drawn as a dashed line. NA (not applicable) indicates that data for the corresponding disease is not available (that is, fewer than 10 adverse reports). Note that for diseases in which most known drugs are proximal to the disease, the efficacy is also higher on average compared with the rest.

Intriguingly, for 165 known drug-disease pairs, the drugs are distant to the disease they are recommended for, indicating that the interactome is unable to explain the drug's effect. The interactome incompleteness can potentially explain the current limitations of network-based drug-disease proximity. Yet, given that the lack of efficacy is the leading reason for failure in drug development^26^, we suspect that the drugs we fail to identify in the proximity of the disease might not be as effective as others. To investigate whether proximity could explain drug efficacy we compile three data sets: (i) Off-label treatments: For each known drug-disease pair, we retrieve the label information from DailyMed and search for the disease in the indication field. If the disease is not mentioned in the indication field we mark this drug-disease association as off-label use (and label use otherwise), resulting in 133 off-label drug-disease associations. (ii) Palliative treatments: For each label use, we check whether the indication field in DailyMed contains any statement referring to the non-causative use of the drug in that disease (for example, manage, relieve, palliate and so on.), yielding 50 palliative drug-disease pairs in which the drug relieves the symptoms of the disease. We mark the remaining 219 drug-disease pairs as non-palliative. (iii) Drug efficacy information: We use side effect and efficacy reports from FDA Adverse Event Reporting System and consider 204 drug-disease pairs associated with at least 10 reports. We count the number of entries for the most commonly observed adverse event and the number of entries reporting that the drug was ineffective. The relative efficacy (RE) score is one minus the ratio of the number of drug ineffective reports to the number of reports with the most common adverse reaction. To confirm that RE captures the palliative nature of drugs, we check the distribution of RE scores of manually curated palliative and the remaining known drug-disease pairs (Fig. 4a), finding that RE scores are significantly lower for palliative drug-disease pairs (one-sided Mann–Whitney *U* test *P*=7.3 × 10^−5^ compared with the distribution of RE scores of non-palliative uses and *P*=7.6 × 10^−4^ compared with that of off-label uses).

Next, we check whether interactome-based proximity can distinguish palliative from non-palliative and off-label drug-disease pairs, observing a significantly lower proximity for drug-disease pairs not described as palliative in DailyMed (Fig. 4b, *P*=4.0 × 10^−5^ and *P*=0.02 for non-palliative and off-label uses, respectively). Given that the description for palliative drug-disease pairs in DailyMed is likely to be incomplete and the non-palliative drug-disease pairs likely include palliative drugs as well, the observed segregation of the palliative and the remaining pairs is striking. Moreover, the lower proximity of off-label uses compared with palliative uses suggests that the current ‘wisdom of the crowd' (off-label treatments recommended by physicians) include promising treatments, most of which likely to be more effective than palliative treatments.

Finally, we explore the distribution of RE scores among proximal and distant drug-disease pairs, finding significantly higher RE scores for proximal drugs (Fig. 4c, *P*=0.04). These findings indicate that proximity is a good measure of a drug's efficacy in the clinic: proximal drugs are more likely to be therapeutically beneficial than distant drugs that usually correspond to palliative treatments.

### Treatment bottlenecks

To illustrate the utility of the developed framework, next we identify diseases in which proximity successfully pinpoints the drugs prescribed for the disease. The percentage of drugs that are proximal to their indicated disease varies substantially over the 78 diseases. When we look at the 29 diseases for which there are at least five known drugs, we see that most drugs used for asthma, Alzheimer's disease (AD), cardiac arrhythmias, cardiovascular diseases, diabetes, epilepsy, hypersensitivity, kidney diseases, liver cirrhosis, systemic lupus erythematosus and ulcerative colitis are proximal to the disease (Fig. 4d, top panel). Similarly, among antineoplastic agents, the drugs used for prostate cancer, breast cancer and lymphoma tend to be proximal to the indicated diseases. Given that AD, breast cancer, heart diseases and diabetes are prevalent in developed countries, they have been at the centre of attention of pharmaceutical companies, potentially explaining the success of the treatments. On the other hand, diseases for which the drugs are distant often involve a substantial inflammatory component, like Crohn's disease, psoriasis and rheumatoid arthritis, suggesting that most of the drugs used in these immune-system-related diseases manage the inflammation or relieve the symptoms of the disease. We also observe that most drugs used in parkinsonian disorders are generally not proximal to the disease. Indeed, for these diseases the RE values are substantially lower compared with the rest of the diseases, confirming that the drugs are more likely to be palliative (Fig. 4d, bottom panel).

**Figure 5:**
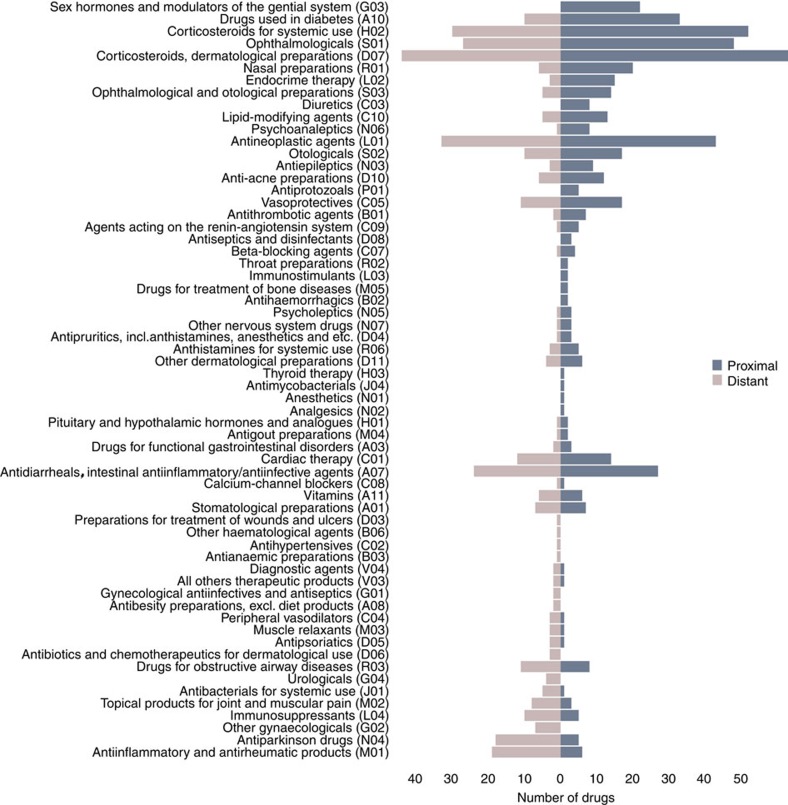
Anatomic therapeutic chemical (ATC) classification of proximal and distant drug-disease pairs. The number of proximal (dark blue) and distant (light brown) drugs in each ATC category among known drug-disease associations. The ATC codes are sorted in descending order with respect to the difference of the number of proximal and distant drugs.

To investigate whether certain groups of drugs are more likely to be proximal to the diseases, we further check their anatomic therapeutic chemical classification (Fig. 5). Again, we find that proximal drugs tend to involve more mechanistic interventions involving the endocrine system and metabolic processes, whereas distant drugs are more enriched in anti-inflammatory and pain relief related categories.

### Uncovering therapeutic links between AD and T2D

Developing effective treatment strategies for diseases requires an understanding of the underlying mechanism of drug action. Next, we show that the network-based proximity can provide insights into the mechanism of action of glyburide and donepezil, two drugs used in T2D and AD, respectively, revealing therapeutic links between these two diseases. Using the pathway information in Reactome database^27^, we identify the pathways that are proximal to these drugs (Methods section). Consistent with the known mechanism of action of glyburide, we find pathways related to the regulation of potassium channels and secretion of insulin (Supplementary Table 1). The drug-pathway proximity also highlights the role of GABA_B_ in regulating G protein receptors during the insulin secretion process.

For donepezil, we find the acetylcholine-related pathway as one of the closest pathways to the drug. Acetylcholinesterase, the known pharmacological action target, catalyses the hydrolysis of acetylcholine molecules involved in synaptic transmission. In addition to the acetylcholine-related pathway, other closest Reactome pathways to donepezil include ‘serotonin receptors', ‘phosphatidylcholine synthesis', ‘adenylate cyclase inhibitory pathway', ‘*IL-6* signalling' and ‘the *NLRP3* inflammasome', thus providing an enhanced view of donepezil's action (Supplementary Table 1). Indeed, a recent study confirms the fundamental role of *NLRP3* in the pathology of AD in mice^28^, offering further insights into how donepezil exerts its therapeutic effect in AD patients. Interestingly, the ‘regulation of insulin secretion by acetylcholine' is among the closest pathways for both drugs. T2D and AD are known to share a common pathology and exhibit increased co-morbidity^29^,^30^. In fact, repurposing anti-diabetic agents to prevent insulin resistance in AD has recently gained substantial attention^31^.

### Dissecting therapeutic benefits from adverse effects

Proximity helps us understand relationships between drugs and diseases and discover novel associations. We first highlight several potential repurposing candidates predicted by proximity among unknown drug-disease pairs. One such candidate is nicotine, a drug originally indicated for ulcerative colitis, which is closer to AD (*z*_c_=−1.2) than its original indication. Indeed, nicotine has recently been argued to improve cognition in people with mild cognitive impairment, a symptom that often precedes Alzheimer's dementia^32^. Not surprisingly, the closest pathways to nicotine are acetylcholine-related pathways such as ‘acetylcholine binding and downstream events', ‘highly calcium permeable postsynaptic nicotinic acetylcholine receptors' and ‘presynaptic nicotinic acetylcholine receptors', closely related to the pathways proximal to donepezil, the AD drug above.

We also find that glimepiride and tolbutamide, two T2D drugs that lower blood glucose by increasing the secretion of insulin, are proximal to cardiac arrhythmia (*z*_c_=−3.6 and *z*_c_ =−2.3, respectively). However, these drugs have recently been suggested to induce adverse cardiovascular events^33^. Therefore, network-based proximity does not always imply that the drug will improve the corresponding disease. To the contrary, some drugs may even induce the disease phenotype by perturbing the functions of the proteins in the proximity of the disease module. To distinguish between a novel treatment and a potential adverse effect, we check the proximity of these drugs to the protein sets predicted to induce the side effects. The proteins inducing a given side effect are predicted based on whether they appear significantly as the targets of drugs with the side effect compared with the targets of drugs without the side effect^34^ (Methods section). Although glimepiride and tolbutamide are proximal to the cardiac arrhythmia disease proteins in the network, they are also proximal to the proteins inducing arrhythmia (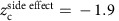 and 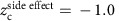, respectively, Supplementary Data 1). In line with earlier findings^33^, proximity indicates that their use by patients with cardiovascular problems requires caution.

Next, we provide interactome-based insights to the drug's action in some recent repurposed uses and clinical failures (Table 1). For instance, we find that proximity can explain why plerixafor, a drug developed against HIV to block viral entry in the cell that failed to meet its end point, is repurposed for non-Hodgkin's lymphoma. We identify that the proximity of plerixafor to the non-Hodgkin's lymphoma disease proteins is *z*_c_=−2.4. On the other hand, when we look at the proximity of tabalumab and preladenant, two drugs failed during clinical trials due to lack of efficacy for systemic lupus erythematosus and parkinson disease, respectively, we observe that these drug-disease pairs are more distant than expected for a random group of proteins in the interactome (*z*_c_>0). Another recent failure is semagacestat, an AD drug that was found to worsen the condition. Semagacestat is proximal to AD proteins in the interactome (*z*_c_=−5.6), indicating that the drug should affect the disease. We are not able to predict the direction of the drug's effect (that is, beneficial or harmful), as there is no protein significantly associated with AD as a side effect. In the case of terfenadine, an antihistamine drug used for the treatment of allergic conditions, however, we find the drug to be proximal to both the cardiac arrhythmia disease proteins (*z*_c_=−2.2) and the proteins predicted to induce arrhythmia (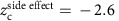) explaining its withdrawal from markets worldwide.

Finally, using proximity, we provide potential repurposing candidates for 2,947 rare diseases retrieved from orpha.net (Supplementary Data 2). Rare diseases are often ignored by pharmaceutical companies due to the small percentage of the population affected and conventional methods are typically unable to offer any candidates. We believe that the proximity-based predictions can provide promising reuses. We note, however, that these predictions need to be validated in the clinic before they can be recommended.

## Discussion

Disease phenotypes are typically governed by defects in multiple genes whose concurrent and aberrant activity is necessary for the emergence of a disease. These disease genes are not randomly distributed in the interactome, but agglomerate in disease modules that correspond to well-defined neighbourhoods of the interactome. Here, we introduced a computational framework to quantify the relationship between disease modules and drug targets using several distance measures that capture the network-based proximity of drugs to disease genes. The systematic analysis of a large set of diseases shows that drugs do not target the disease module as a whole but rather aim at a particular subset of the disease module. Moreover, the impact of drugs is typically local, restricted to disease proteins within two steps in the interactome.

Proximity provides insights into the drug mechanism of action, revealing the pathobiological components targeted by drugs and increases the applicability and interpretability for repurposing existing drugs. We find that if a drug is proximal to the disease, it is more likely to be effective than a distant drug. We argue that for diseases in which the drugs are distant, the drugs alleviate the symptoms of the disease. We observe that off-label treatments are at least as effective as palliative uses mentioned in the label, providing an interactome-level support for off-label uses of drugs. We use adverse event reports collected by FDA to offer evidence that many disorders involving immune response are indeed targeting the disease symptoms. We also demonstrate several proof-of-concept examples in which proximity successfully predicts both the therapeutic and the adverse effects of known drugs.

We also used proximity to define similarity between two drugs and showed that proximity performed at least as good as existing similarity-based approaches and covered larger number of drug-disease associations. Nevertheless, similarity-based methods can only predict drugs for diseases that already have a drug, therefore are ineffective for drugs that do not share any target with existing drugs or for diseases without known drugs, as it is the case for many rare diseases. Furthermore, these approaches typically do not offer a mechanistic explanation of why a drug would (or would not) work for a disease. On the other hand, proximity enables us to suggest candidate drugs to be repurposed in rare diseases.

Given the limitations of the current interactome maps, from incompleteness to investigative biases, we have explored how the number and the centrality of drug targets and disease proteins influence their network-based proximity. We find that proximity is not biased with respect to neither the number of targets a drug has nor their degrees. Thus, proximity corrects a common pitfall in existing studies that do not account for the elevated number of interactions of drug targets. Moreover, we find that the integrated interactome used in this study captures the therapeutic effect of drugs better than both functional associations from STRING database^35^ and protein interactions from high-throughput binary screens^13^, two interactome maps widely used in the literature (Supplementary Table 2). A potential drawback of proximity is that it relies on known disease genes, drug targets and drug-disease annotations, all of which are known to be far from complete. Although we ensure that the annotations used in the analysis are of high quality using various control data sets (Supplementary Table 2 and Supplementary Note 6) the coverage of our analysis can be increased as more data become available. Furthermore, the directionality of the drug's predicted effect (for example, whether it is beneficial or harmful) depends on the characterization of the proteins inducing the disease, information that is currently limited to only a small subset of the diseases.

Overall, our results indicate that network-based drug-disease proximity offers an unbiased measure of a drug's therapeutic effect and can be used as an effective and holistic tool to identify efficient treatments and distinguish causative treatments from palliative ones. While proximity can provide a systems level explanation towards the drug's effect via quantifying the separation between the drug and the disease in the interactome, understanding the therapeutic effect of drugs at the individual level (that is, patients with different genetic predisposition) requires incorporating large scale patient level data such as electronic health records and personal genomes and remains the goal of future work in this area. It would also be interesting to extend the analysis presented here to drug combinations, in which the proximity of the targets of the combination is likely to be different than the average proximity of the drugs individually, potentially giving insights into the synergistic effects.

## Methods

### Drug, disease and interaction data sets

The disease-gene data relied on Menche *et al.*^12^, defining diseases using MeSH. Disease-gene associations were retrieved from OMIM and GWAS catalog using UniProtKB (ref. 36) and PheGenI (ref. 37), respectively. Only the genes with a genome-wide significance *P* value <5.0 × 10^−8^ were included from PheGenI. We used only the diseases for which there were at least 20 known genes in the interactome. This cutoff based on number of disease genes ensures that the diseases are genetically well characterized and are likely to induce a module in the interactome^12^. For each disease, we looked for information on FDA approved drugs in DrugBank (downloaded on July 2013) and matched 79 of these diseases with at least one drug using MEDI-HPS (ref. 17; using *MEDI_01212013_UMLS.csv* file) and Metab2Mesh (ref. 18; retrieved from metab2mesh.ncibi.org on June 2014). MEDI-HPS contains drug-disease associations compiled from RxNorm, MedlinePlus, SIDER (ref. 21) and Wikipedia. We considered a drug to be indicated for a disease if and only if the and there was a strong association based on text-mining in Metab2Mesh (*Q* value<1.0 × 10^−8^), yielding 337 drugs. We excluded 99 drugs that either had no known targets in the interactome or had the same targets as another drug used for the same disease, resulting in a total of 238 unique drugs and 384 targets. Note that we only considered the pharmacological targets (‘Targets' section in DrugBank), excluding the enyzmes, carriers and transporters that were typically shared among different drugs. To ensure the quality of the drug-disease associations, we downloaded label information for each of these drugs from DailyMed (dailymed.nlm.nih.gov) and checked the indication field (Supplementary Data 1). For each drug, we first matched the drug name (and synonyms if there was no match) in the Rx_norm_mapping file and fetched the drug's structured product labelling id(s). We then queried DailyMed using the structured product labelling id. We noticed that Felbamate was incorrectly annotated to be used for aplastic anaemia in MEDI-HPS while it was a clear contraindication for this disease. Accordingly, we removed aplastic anaemia from the analysis as there were no other drugs associated with it. For calculating enrichment of proximal drug-disease pairs in clinical trials, we retrieved information on the drugs and the diseases they were tested for from clinicaltrials.gov.

We took the human protein–protein interaction (PPI) network compiled by Menche *et al.*^12^ that contained experimentally documented human physical interactions from TRANSFAC^38^, IntAct^39^, MINT^40^, BioGRID^41^, HPRD^42^, KEGG^43^, BIGG^44^, CORUM^45^, PhosphoSitePlus^46^ and a large scale signalling network^47^. We used the largest connected component of the interactome in our analysis, consisting of 141,150 interactions between 13,329 proteins. ENTREZ Gene IDs were used to map disease-associated genes to the corresponding proteins in the interactome. The interactome and disease-gene association data is provided as a supplementary data set in Menche *et al.*^12^ The drug-target interactions are provided in Supplementary Data 3.

To calculate proximity of drugs for rare diseases, we downloaded 3,323 diseases and genes associated with them from orpha.net. For each disease gene, we mapped the Uniprot ID to Gene ID using the external reference field in the XML file and filtered for only the diseases that had at least a known disease protein in the interactome, yielding 2,947 diseases. We then calculated the proximity between each FDA approved drug and the disease. The drugs that did not have any targets in the interactome or that had the same targets as another drug were excluded.

### Network-based proximity between drugs and diseases

The proximity between a disease and a drug was evaluated using various distance measures that take into account the path lengths between drug targets and disease proteins. Given *S*, the set of disease proteins, *T*, the set of drug targets and *d*(*s*,*t*), the shortest path length between nodes *s* and *t* in the network, we define:


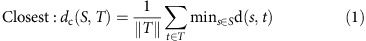



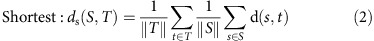



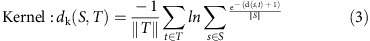



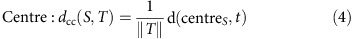


where centre_*S*_, the topological centre of *S* was defined as


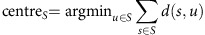


in case the centre_*S*_ is not unique, all the nodes are used to define the centre and the shortest path lengths to these nodes are averaged.


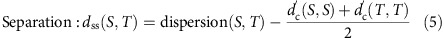


where 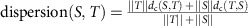 and 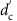 is the modified closest measure in which the shortest path length from a node to itself is infinite.

To assess the significance of the distance between a drug and a disease (*T*,*S*), we created a reference distance distribution corresponding to the expected distances between two randomly selected groups of proteins matching the size and the degrees of the original disease proteins and drug targets in the network. The reference distance distribution was generated by calculating the proximity between these two randomly selected groups, a procedure repeated 1,000 times. The mean *μ*_*d(S,T)*_ and s.d. *σ*_*d(S,T)*_ of the reference distribution were used to convert an observed distance to a normalized distance, defining the proximity measure:


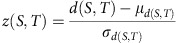


due to the scale-free nature of the human interactome, there are few nodes with high degrees. To avoid repeatedly choosing the same (high degree) nodes during the degree-preserving random selection, we used a binning approach in which nodes within a certain degree interval were grouped together such that there were at least 100 nodes in the bin. Accordingly, each bin *B*_*i,j*_ was defined as *B*_*i,j*_={*u*∈*V*|*i*≤*k*_*u*_<j} containing the nodes with degrees *i* to minimum possible *j* such that ||*B*_*i,j*_||≥100.

### Area under ROC curve and optimal proximity cutoff analysis

We used AUC to evaluate how well the distance measures discriminated known drug-disease pairs from unknown drug-disease pairs. Given a set of known drug-disease associations (positive instances) and a set of drug-disease couplings in which the drug is not expected to work on the disease (negative instances), the true positive rate and false positive rate were calculated at different thresholds to draw the ROC curve. The area under this curve was computed using the trapezoidal rule. While known drug-disease associations can be used as positive control, defining the negative control (drugs that have no effect on a disease) is not straightforward. As a proxy, we assumed that all unknown drug-disease associations were negatives, thereby ignoring potential positive cases among the unknown associations. Furthermore, to control for the size imbalance of known and unknown drug-disease associations, we randomly chose 402 pairs among unknown drug-disease associations and used them as negatives in the AUC calculation. We repeated this procedure 100 times and used the average of the AUC values to compare the distance measures (Supplementary Fig. 4). Again, the AUC values were consistent with what we observed using all unknown drug-disease pairs as negatives, pointing out the robustness of drug-disease proximity against negative data selection. In both models, the closest measure discriminates best the known drug-disease associations from the random drug-disease associations, as it was observed using all unknown drug-disease pairs as negatives.

To find the optimal network-based proximity threshold (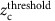) for which a drug was more likely to work on (proximal to) a certain disease, we used proximity versus sensitivity and specificity curves. Sensitivity corresponds to the percentage of the positive (known) drug-disease associations that are found proximal among all positive drug-disease associations. Specificity corresponds to the percentage of the negative (unknown or random) drug-disease associations that are not proximal among all negative drug-disease associations. Accordingly, the network-based proximity threshold, 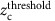, giving both high coverage (assessed by sensitivity) and low number of false positives (assessed by 1-specificity) was defined as the value at which the sensitivity and specificity curves intersected (Supplementary Fig. 4). In our analysis, we set 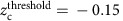, that is, a drug was defined to be proximal to a disease if the proximity between them was ≤−0.15. To ensure the robustness of 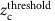, we repeated the analysis on two other data sets and showed that the 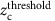 value was similar (Supplementary Note 5). In addition to sensitivity and specificity, we provide *F*-score (harmonic mean of precision and sensitivity) measures at different proximity cutoffs. A different cutoff value can be used to define proximity depending on the desired coverage and false positive rate.

### Evaluating the therapeutic effect of drugs

We annotated the drug-disease associations based on whether the label information in DailyMed contained the drug-disease association given in MEDI-HPS. Accordingly, we marked 269 drug-disease associations appearing in the label as label use and the remaining 133 drug-disease associations as off-label use (Supplementary Data 1). We also looked for statements referring to the non-causative use of the drug in that disease in the DailyMed indication field. We specifically searched for sentences containing the following keywords and their variations: ‘palliative', ‘symptomatic' and ‘signs and symptoms'. We required that the disease the drug was used for was unambiguously mentioned in the indication field. This data set contained 50 of 402 known drug-disease pairs in which the drug was used to manage the signs and symptoms of the disease (Supplementary Data 1).

We compiled drug efficacy information using the adverse event reports submitted to FDA Adverse Event Reporting System. A report lists the patient reaction for a given drug and disease including ‘pain', ‘nausea' and ‘drug ineffective' among many other reactions. We used openFDA Application Programming Interface (api.fda.gov/drug) to retrieve the adverse reaction information and considered only 204 drug-disease pairs for which there were at least 10 adverse event reports for the most common adverse reaction. We counted the number of reports containing the ‘drug ineffective' reaction (*n*_inefficient_) and derived a score, RE, by comparing it with the number of most occurring reaction (*n*_top_) for that drug-disease pair. The RE is defined as the complement to one of relative inefficacy, where relative inefficacy is the ratio of the number of ‘drug ineffective' reports to the number of most common adverse event reports. Hence,


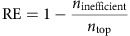


The RE takes values between 0 (poorest efficacy, ‘drug ineffective' reports are the most common reports) and 1 (there is no ‘drug ineffective' report associated with this drug-disease pair). For instance, among the reports containing atorvastatin and arteriosclerosis, ‘myalgia' was the most common reaction with 13 occurrences and there were two reports containing ‘drug ineffective', yielding RE=0.85. When multiple drugs are reported in the same entry, the observed reactions may not be due to all drugs. Nevertheless RE still provides a reasonable proxy for the efficacy of the drug. In addition to the drug names provided in DrugBank, synonyms and brand names were queried through the API and the query returning the most results was chosen to represent the drug and used in further queries fetching reactions. The disease names were also modified to match the names used in the openFDA data set.

### Network-based pathway and side-effect proximity analysis

To identify the biological pathways affected by a drug in the human interactome, we used the closest measure to quantify the proximity between drugs and pathways. The drug-pathway proximity is the normalized distance calculated between the drug targets and proteins belonging to a given pathway. Similar to drug-disease proximity, randomly selected protein sets matching the original protein sets in size and degrees were used to calculate the mean and the s.d. for the *z*-score calculation. We used all Reactome pathways provided in MsigDB (ref. 48) that had at most 50 proteins (as larger pathways tend to describe broader biological processes) and ranked all the pathways with respect to their proximity to a given drug. We report the proximity values between the drugs and the diseases, pathways, and side effects in Supplementary Data 1.

To check whether a drug was proximal to the proteins inducing certain side effects, we first defined the protein sets inducing side effects and then calculated the network-based proximity of drug targets to these proteins (same as we did for disease and pathway proteins). The side-effect proteins were identified using a Fisher's test-based enrichment analysis^34^. Accordingly, for each side-effect reported for at least five drugs in SIDER^21^ and for each target of these drugs, we counted the number of drugs that the side effect and drug-target appeared together as well as the number of drugs in which they appeared individually (only side effect or only drug) and did not appear at all together. We then corrected the two-sided *P* value for multiple hypothesis testing using Benjamini and Hochberg's method to decide whether a drug-target induced a certain side effect. For each side effect, the targets <20% false discovery rate were predicted to induce the side effect. For each of the 78 diseases in the data set, we manually mapped the MeSH disease terms to SIDER side-effect terms where available (58 out of 78 diseases) and used 17 side effects that had at least one predicted protein. The proximity values between the drugs and these side effects are given in Supplementary Data 1. The targets inducing these side effects are given in Supplementary Data 3. In addition to 238 FDA approved drugs used in the analysis, we provide the drug disease and drug side effect proximities of 45 withdrawn drugs that have at least one corresponding target in the interactome (Supplementary Data 1).

### Statistical tests and code availability

We used Fisher's exact test and two-sided *P* values associated with it to evaluate the strength of the enrichment of proximal drug-disease pairs among known and unknown drug-disease pairs. The alpha value for the significance of *P* values was set to 0.05. For assessing difference between means of distribution of RE values, one-sided Mann–Whitney *U* test was used with the same alpha value as before. The alternative hypotheses for the one-sided test were (i) the palliative drugs were expected to have lower RE values, (ii) the palliative drugs were expected to have larger proximity values and (iii) the proximal drugs were expected to have higher RE values. We used R (r-porject.org) for statistical tests and data visualization and Python (python.org) to parse various data sets and to calculate drug-disease proximity (see toolbox package located at github.com/emreg00/toolbox).

## Additional information

**How to cite this article**: Guney, E. *et al.* Network-based *in silico* drug efficacy screening. *Nat. Commun.* 7:10331 doi: 10.1038/ncomms10331 (2016).

## Supplementary Material

Supplementary Information Supplementary Figures 1-4, Supplementary Tables 1-2, Supplementary Notes 1-6 and Supplementary References

Supplementary Data 1 Drug-disease annotations and proximity values of drugs to diseases, pathways and side effects

Supplementary Data 2 Drug proximity values for rare diseases

Supplementary Data 3 Drug-target interactions and targets predicted to induce diseases.

## Figures and Tables

**Table 1:** Proximity values for several repurposed and failed drugs.

**Drug**	**Description**	**Phenotype**	**Proximity (*****z*****)**
Repurposed uses			
Plerixafor	Repurposed to treat non-Hodgkin's lymphoma	Non-Hodgkin's lymphoma	−2.4
Ropinirole	Repurposed to treat restless legs syndrome	Restless legs syndrome	−1.1
Sildenafil	Repurposed to treat erectile dysfunction	Erectile dysfunction	−1.0
			
Metadata based observations^49^			
Drospirenone	Confer protection against endometrial cancer	Endometrial cancer	−1.1
Levonorgestrel	Confer protection against endometrial cancer	Endometrial cancer	−1.6
			
Failures due to lack of efficacy			
Tabalumab	Showed lack of efficacy for systemic lupus erythematosus	Systemic lupus erythematosus	1.8
Preladenant	Discontinued trials for Parkinson due to lack of improvement compared with placebo	Parkinson's disease	0.2
Iniparib	Failed to achieve improvement while being tested for squamous non-small-cell lung cancer	Squamous cell cancer	0.0
			
Failures due to adverse effetcs			
Semagacestat	Failed trials due to worsening AD	AD	−5.6
Terfenadine	Withdrawn due to inducing cardiac arrhythmia	Cardiac arrhythmiaArrhythmia (side effect)	−2.2−2.6
