# Supplementary material for: Network-based in silico drug efficacy screening
Source: Nat Commun. 2016 Feb 1;7:10331. doi: 10.1038/ncomms10331 (PMC4740350; doi:10.1038/ncomms10331)
Supplement: Supplementary Information — Supplementary Figures 1-4, Supplementary Tables 1-2, Supplementary Notes 1-6 and Supplementary References [file ncomms10331-s1.pdf]

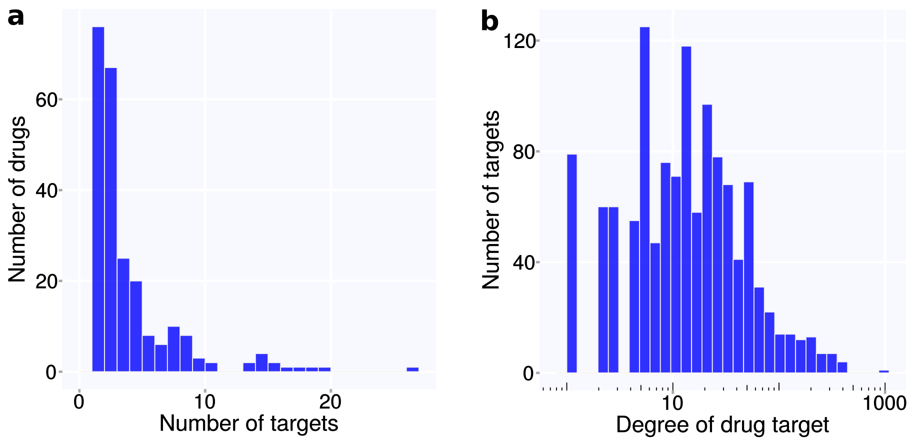

Supplementary Figure 1: Drug target and degree information. Histograms of **(a)** number of drug targets per drug (the mean is 3.5 and the median is 2) and **(b)** degree of the targets in the interactome (the mean is 28.6 and the median is 12). The drug target with the highest degree is GRB2 (with 872 interactions).

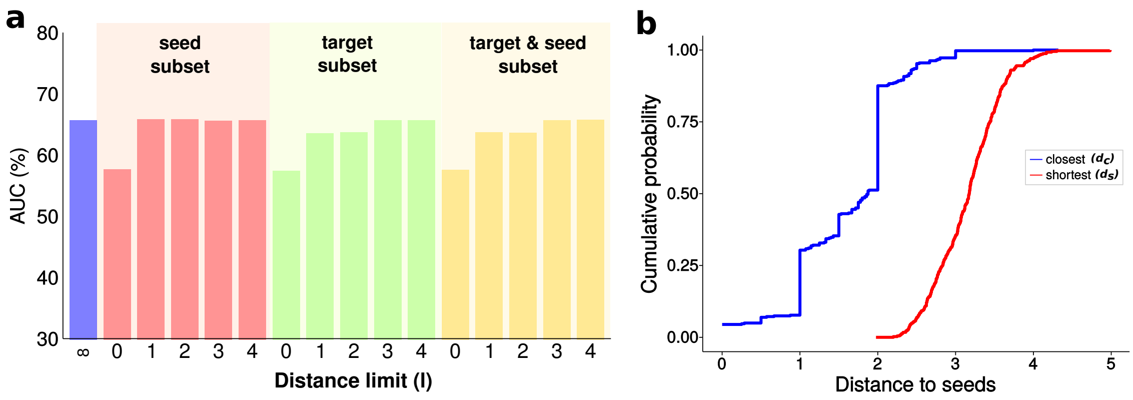

Supplementary Figure 2: Prediction performance of the closest method using only a subset of targets or disease proteins. **(a)** AUC values using a subset of disease proteins (seeds), drug targets and both drug targets and seeds in which the subset is defined by the distance from drug targets to disease proteins (and vice versa) using the closest measure. In subset l_i, a disease protein (drug target) is included in the set if it is at most i steps away from the closest drug target (disease protein). **(b)** The plot shows the cumulative probability distribution of closest and shortest distances from drug targets to disease proteins.

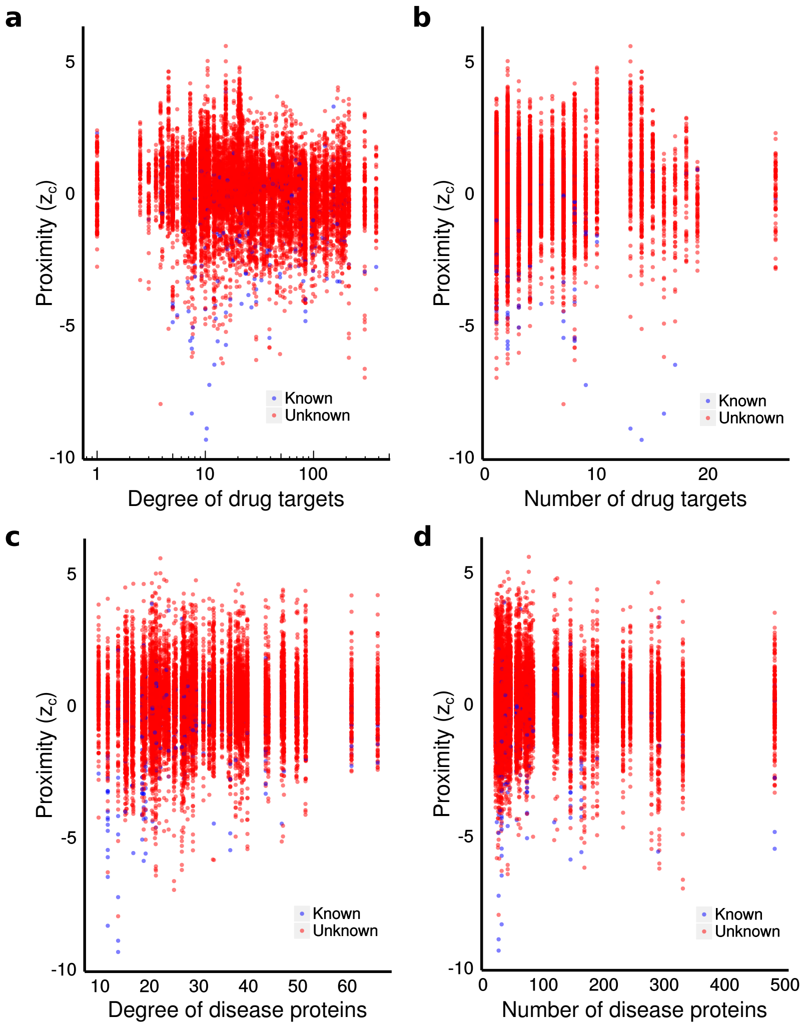

Supplementary Figure 3: Proximity versus number and degrees of drug targets and disease proteins. The plots show the proximity of known (blue) and unknown (blue) drug-disease pairs versus **(a)** the degree of drug targets, **(b)** the number of drug targets, **(c)** the degree of disease proteins, and **(d)** the number of disease proteins.

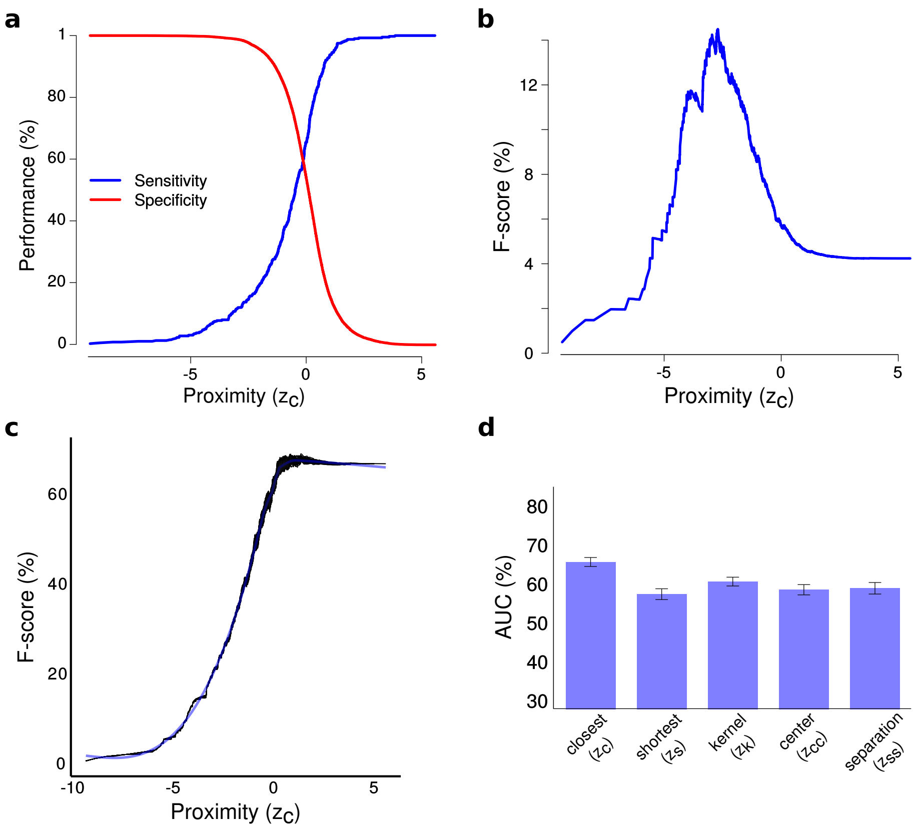

Supplementary Figure 4: Assessing prediction performance of proximity. (a) Plot shows Sensitivity and Specificity curves over different proximity values. The proximity has both fair true positive rate (Sensitivity) and true negative rate (Specificity) at $z_c = -0.15$ (the point where the curves meet). **(b)** F-score (harmonic mean of Precision and Sensitivity) versus proximity using all unknown drug-disease associations as negatives. The low f-score is due to the positives constituting a small portion of the all drug-disease associations and the negatives including potential “positives” (repurposing opportunities or drugs worsening the disease condition), giving rise to low Precision. **(c)** F-score versus proximity using 100 groups of randomly sampled unknown drug-disease associations as negatives. Each group contains the same number of negative instances as positive instances (known drug-disease pairs). The blue line shows the average F-score over 100 random groupings. The balanced number of positive and negative instances yields better F-scores. **(d)** The AUC values of distance measures using 100 groups of randomly sampled unknown drug-disease associations as negatives. The AUC values are consistent with the values observed using all unknown pairs as negatives, closest measure outperforming the remaining measures. The lines show standard error over 100 different groupings of the unknown drug-disease associations.

Supplementary Table 1: **Top 10 proximal pathways for donepezil and glyburide.**

Pathway	n	z
**Donepezil**		
synthesis of phosphatidylcholine	11	-3.3
serotonin receptors	11	-3.3
adenylate cyclase inhibitory pathway	13	-2.2
IL-6 signaling	10	-2.1
the NLRP3 inflammasome	11	-2.1
regulation of insulin secretion by acetylcholine	10	-2.1
regulation of IFN gamma signaling	13	-2.0
growth hormone receptor signaling	24	-2.0
advanced glycosylation endproduct receptor signaling	13	-2.0
ADP signalling through P2RY12	21	-1.9
**Glyburide**		
inwardly rectifying K^+ channels	30	-9.0
ABC family proteins mediated transport	22	-8.5
Inhibition of voltage gated Ca^{+2} channels via G beta gamma subunits	25	-4.3
$GABA_B$ receptor activation	38	-4.1
regulation of insulin secretion by acetylcholine	10	-3.3
Na^+/Cl^- dependent neurotransmitter transporters	9	-3.3
trafficking of GluR2 containing AMPA receptors	15	-2.8
amine compound SLC transporters	14	-2.8
amine ligand binding receptors	31	-2.6
gap junction degradation	10	-2.5

Supplementary Table 2: **Prediction performance of the drug-disease proximity (z_c) using various data sets.**

**Data set**	**Number of diseases**	**Number of drugs**	**Number of drug-disease pairs**	**AUC (%)**
Original	78	238	402	65.7
Protein interactions				
Binary interactome	50	129	226	58.3
STRING	77	233	396	61.3
Disease-gene associations				
OMIM	35	114	155	71.2
GWAS	44	157	260	60.2
Drug-target associations				
STITCH	73	212	359	64.8
Disease-drug associations				
NDF-RT	61	160	233	66.2
KEGG	16	74	76	71.3
Original data set filtered using				
Diseases with at least one protein	304	462	1192	58.6
Diseases excluding broader MeSH term	53	205	282	67.2
Drugs with at least three targets	49	95	144	64.6
Drugs whose targets are not disease proteins	76	227	384	64.5

Supplementary Note 1: Drugs target two-step neighborhood of the disease genes

To pinpoint drug-disease associations even when the target is not a disease protein, we defined the drug-disease proximity using several network-based distance measures. We observe that the closest measure captures the drug-disease proximity better than the remaining measures, suggesting that drug targets do not necessarily have to be close to all the proteins in the disease module. Motivated by this observation, we test the performance of the network-based proximity using only (*i*) disease proteins at most l steps away from a drug target (seed subset), (*ii*) the drug targets at most l steps away from a disease protein (target subset), (*iii*) the drug target and disease protein pairs that are at most l steps away from each other (target-seed subset). Note that the seed and target subset approaches are not symmetric: Given a set of drug targets $T = \{t_1, t_2\}$ and a set of disease proteins $S = \{s_1, s_2\}$, say while the closest disease protein to the drug target t_1 is s_1, the closest drug target to s_1 might be t_2 but not t_1. To restrict the distance calculation to a given distance l, we first calculate the shortest path distances between each pair of drug target (t_i) and disease protein (s_j), sort these distances and then consider only the pairs (t_i, s_j) for which $d(t_i, s_j) \leq l$.

Through exhaustive search of parameter space ($l \in \{0, 1, 2, 3, 4\}$), we find that the AUC does not change significantly after $l = 2$ (Supplementary Fig. 2a). Furthermore, the AUC at $l = 2$ is comparable to AUCs when all disease genes or all drug targets are considered. Indeed, the distribution of distances between drug targets and disease proteins among

known drug-disease pairs shows that 90% of the drugs have a known disease protein within two steps (Supplementary Fig. 2b). This suggests that most drugs exert their therapeutic effect on the disease proteins that are at most two steps away.

Supplementary Note 2: Proximity does not depend on the number and degree of drug targets and disease proteins

Several factors such as the number and degree of the drug targets and disease proteins can influence the discriminatory performance of the drug-disease proximity measure. Drugs with more targets or whose targets are more central are expected to be closer to a disease protein (and vice versa). To check whether proposed proximity measure is biased towards such drugs, we plot proximity versus number of drug targets and degree of drug targets among all possible drug-disease associations. We find that both number of targets of a drug and the average degree of the drug's targets show almost no correlation with proximity (Spearman's rank correlation coefficient, Suuplementary Fig. 3a-b, $\rho = 0.08$, $P = 9.6 \times 10^{-31}$ and $\rho = -0.10$, $P = 1.9 \times 10^{-46}$, respectively). Similarly, the drug-disease proximity is not correlated with neither the number of disease proteins (Supplementary Fig. 3c-d, $\rho = -0.01$, $P = 0.12$), nor with the average degree of disease proteins ($\rho = 0.03$, $P = 3.1 \times 10^{-5}$).

Supplementary Note 3: Proximity and drug similarity based repurposing

Drug-drug similarity is often used to predict a novel use for a given drug. The similarity between two drugs is usually defined based on sharing chemical structure[1], targets[1, 2, 3],

functional annotations (of the targets)[1] or side effects[4, 1] as well as shortest path distance between targets in the interactome[1]. Accordingly, given two drugs X and Y with targets T_X and T_Y, we calculate:

(i) the interactome-based distance between the targets of X and Y:

$$\delta_{\text{target PPI}}(X, Y) = e^{-l(X,Y)}$$

where $l(X, Y)$ is defined as

$$l(X, Y) = \frac{\sum_{u \in T_X, v \in T_Y} d(u, v)}{\|T_X \cup T_Y\|}$$

and $d(u, v)$ denoting the shortest path distance between proteins (u, v) in the interactome. Accordingly, two drugs X and Y are similar if their targets are close to each other in the interactome. For defining proximity-based similarity, we use $z_c(X, Y)$ instead of $l(X, Y)$.

(ii) the ratio of common drug targets of X and Y:

$$\delta_{\text{target}}(X, Y) = \frac{\sum_{t \in T_X \cap T_Y} w_t}{\|T_X \cup T_Y\|}$$

where w_t, the disease-specificity of each target (the number of diseases for which a drug with target t is used), is given by

$$w_t = \frac{1}{\sum_{i \in D} I_i^t}$$

with D being all the diseases analyzed in this study and I_i^t being an indicator variable defined as

$$I_i^t = \begin{cases} 1, & t \text{ is targeted by a drug used for disease } i \\ 0, & \text{otherwise} \end{cases}$$

That is, the similarity between drugs X and Y is based on the number and disease-specificity of their shared targets. Note that if $w_t = 1$ for all targets, the similarity reduces to the Jaccard index of the targets of X and Y ignoring whether the targets are disease-specific or not.

(iii) chemical similarity between X and Y:

$$\delta_{\text{chemical}}(X, Y) = \frac{\|F_X \wedge F_Y\|}{\|F_X \vee F_Y\|}$$

where F_X, F_Y are 2D SMILES fingerprints of drug X and Y, respectively. That is, the chemical similarity of drugs X and Y is defined as the Tanimoto index of the SMILES fingerprints of X and Y. We first converted the SMILES fingerprints to aromatic form and then calculated Tanimoto index using Indigo Python toolkit (lifescience.opensource.epam.com/indigo).

(iv) the ratio of GO terms shared among the targets of X and Y:

$$\delta_{\text{GO}}(X,Y) = \frac{\sum\limits_{m \in M_X \cap M_Y} w_m}{\|M_X \cup M_Y\|}$$

where M_X and M_Y are the set of GO molecular function terms annotated for T_X and T_Y, respectively and w_m is the disease-specificity of each common GO term m calculated based on the number of diseases m appears among the targets of the drugs used for each disease. Thus, $\delta_{GO}(X,Y)$ gives the functional similarity of drugs X and Y as the common disease-specific molecular function GO terms. Gene annotations were downloaded from GO web page (geneontology.org/page/downloads) in July, 2013.

(v) the ratio of common side effects of X and Y:

$$\delta_{\text{side effect}}(X,Y) = \frac{\sum\limits_{e \in E_X \cap E_Y} e_m}{\|E_X \cup E_Y\|}$$

where E_X and E_Y are known side effects of drugs X and Y, respectively and we is the disease-specificity of each common side effect e calculated based on the number of diseases for which a drug with e exists. The side effects of drugs are retrieved using SIDER database[5]. The drugs are mapped to each other via the PubChem identifiers provided in DrugBank and SIDER databases.

(vi) the perturbation profile similarity of X and Y:

$$\delta_{\text{LINCS}}(X,Y) = \frac{\|P_X \cap P_Y\|}{\|P_X \cup P_Y\|}$$

corresponding to the ratio of common differentially regulated genes in the perturbation profiles of X and Y in LINCS database located at lincsproject.org where P_X and P_Y are the gene sets that are differentially expressed upon perturbation by drugs X and Y, respectively. The differentially expressed 100 landmark genes (lm100) upon drug perturbations were retrieved using LINCS API in June, 2014 (api.lincscloud.org) and in case of multiple perturbations for the same drug (i.e. multiple cell lines, perturbation times or dosages), the perturbations resulting in highest similarity ($\delta_{\text{LINCS}}(X,Y)$) are used.

Although predicted side effects, drug targets or disease-disease similarity information can increase the coverage of these methods, their use is likely to have a significant impact on the prediction performance due to the limited reliability of available prediction methods. Furthermore, it is not possible to discover novel drugs whose targets have not been explored for a particular disease or to find drugs that do not have a certain (e.g., undesired) side effect because of the dependence on the existing drug and disease information. Drug-disease proximity overcomes these limitations, as it does not depend on the existing knowledge of drug-disease associations.

Supplementary Note 4: Comparing proximity to gene expression based repurposing

To identify drugs that can potentially account for the gene expression changes induced by diseases, recent studies proposed using correlation of gene expression between the disease state and after treatment with drug[6, 7]. The premise of these studies is to find drugs whose

perturbation profiles are anti-correlated with the genes perturbed in the disease such that the treatment with the drug can revert the expression changes in the disease state. That is, for instance, if a gene is over-expressed in the disease condition, the goal is to find a drug that yields the under-expression of that gene. We test this hypothesis using Drug versus Disease (DvD) R package[8] to correlate drug and disease gene expression profiles from public microarray repositories. DvD provides the precalculated reference ranked gene lists based on differential expression from disease states in Gene Expression Omnibus (GEO, ncbi.nlm.nih.gov/geo) and drug perturbations in Connectivity Map[9] (DrugVsDiseasedata and cMap2data R data packages, respectively). In DvD, disease profiles are defined for 45 diseases based on various data sets in GEO and drug profiles are defined by merging multiple samples for the same compound for 1309 compounds in Connectivity Map version 2[10, 8]. The 200 significantly differentially expressed genes (top and bottom 100 genes in the ranked lists) are used to calculate an enrichment score based on Kolomgorov-Smirnov statistic (i.e. calculateES function in the R package), corresponding to the strength of the anti-correlation of drug and disease profiles. DvD had information for 72 drugs and 14 diseases in our data set covering 95 out of 402 known drug-disease pairs and 1,885 out of 18,162 unknown pairs.

Supplementary Note 5: Robustness of drug-disease proximity threshold

To define proximal and distant drug-disease pairs, we examine the coverage of known and unknown drug-disease associations at various thresholds and choose the threshold, $z^{\text{threshold}}$, that gives both high coverage and low false positive rate (Sensitivity and 1-

Specificity, respectively) identified by the threshold for which Sensitivity and Specificity have both high values. We use ROCR package[11] to calculate the Sensitivity and Specificity values and then find the cutoff for which these values are equally high (i.e. the difference between the two values are within $|\Delta| \leq 1\%$). For the original data set used in the analysis, $z^{\text{threshold}} = -0.15$ with a Sensitivity of 59% and Specificity of 60%.

We confirm that the selected interactome-based proximity threshold does not change significantly by repeating our analyses using drug-disease associations from (*i*) NDF-RT and (*ii*) KEGG. On both data sets, we find that the threshold is similar to that of the original data set ($z^{\text{threshold}}_{\text{NDF-RT}} = -0.10$ and $z^{\text{threshold}}_{\text{KEGG}} = -0.07$, respectively). We also check the enrichment of known drug-disease pairs among proximal and distant drug-disease pairs to ensure that our findings on the relationship between the proximity and a drug's therapeutic effect generalizes over different data sets. Consistent with the original analysis we find that drugs proximal to a disease are at least 2 times more likely to be effective on that disease in both data sets (Fisher's exact test, $OR = 2.2, P = 4.8 \times 10^{-9}$ using NDF-RT and $OR = 3.0, P = 4.8 \times 10^{-6}$ using KEGG).

Supplementary Note 6: Controlling for data quality

Data incompleteness and study bias pose substantial challenges in the systematic analysis and interpretation of biological data. Current literature provides a snapshot of drugs known to be effective in several diseases, known drug targets, disease genes and protein-protein interactions. To make sure that the drug, disease and interaction data sets used in our analysis constitute an accurate representation of the state-of-the-art, we test the

performance of drug-disease proximity measure across different data sets (Supplementary Table 2).

To evaluate the effect of the underlying network on proximity, in addition to the integrated human interactome (PPI), we use the binary human interactome compiled from high-quality yeast two-hybrid interaction detection screens and literature[12] (Lit-BM-13 and HI-II-14 at interactome.dfci.harvard.edu/H_sapiens/host.php). The binary interactome covers 7,544 proteins and 24,202 interactions between them, thus it is much smaller than PPI. The AUC corresponding to discrimination of known and unknown drug-disease pairs drops significantly, indicating that the coverage of the interactome has a significant effect on the drug-disease proximity. Though binary assays provide systematic high-quality data, their coverage is limited[13]. To counterbalance this limitation, we use a functional association network from STRING database [14] containing interactions with a confidence score 700 or higher. The STRING network has 16,086 proteins and 314,656 interactions, more than double the number of interactions in the PPI network. Yet, the AUC is slightly higher than that of binary interactome, suggesting that both the quality and the coverage of the protein interaction data have a significant impact on the proximity between drugs and diseases.

Next, we assess the effect of disease annotations on drug-disease proximity by using only disease gene information from either the OMIM database or the GWAS Catalogue. The AUC using only OMIM data is higher than the original AUC (using both OMIM and GWAS genes), whereas the AUC using only GWAS data is substantially lower. However, among 78 diseases in the original data set, there are 43 diseases that have no associated genes in

OMIM database. Therefore, using the data from both OMIM and GWAS substantially increases the coverage of the diseases.

To account for the limitations of drug-target association data[15], we also use drug target information from STITCH database[16] that integrates known and predicted drug target associations based on evidence in the literature. For each drug, the proteins with confidence score greater than 700 are considered to be targeted by the drug in addition to the targets provided in DrugBank. This data set contains 2,244 distinct targets for 212 drugs. The median number of targets per drug using STITCH is significantly higher (15 targets per drug vs. 2 targets per drug using DrugBank). Nonetheless, the AUC is slightly lower, suggesting that quality of drug-target information is at least as important as the coverage.

To make sure that the drug-disease annotations used in our analysis is of high confidence, in addition to MEDI-HPS, we collect drug-disease assocations from National Drug File - Resource Terminology (NDF-RT)[17] and Kyoto Encyclopedia of Genes and Genomes (KEGG)[18]. We retrieve the drug-disease associations using NDF-RT (rxnav.nlm.nih.gov/NdfrtAPIs.html) and KEGG (rest.kegg.jp) REST APIs, respectively. In NDF-RT, a drug is considered to be indicated for a disease if and only if the drug's NDF-RT entry contained a "may treat" relationship with the disease. Similar to the drug-disease associations used in the original analysis, we filter these drug-disease associations using Metab2Mesh[19] (q-value $< 1 \times 10^{-8}$). The AUC is considerably higher using drug-disease associations from KEGG, suggesting that the annotations in KEGG tend to be more reliable. Nonetheless, the number of drugs and diseases included in the analysis is significantly lower compared to

the annotations from MEDI-HPS. Hence, MEDI-HPS offers a good compromise between accuracy and coverage of drug-disease associations, allowing us to analyze the most number of drugs and diseases.

We also examine the AUC value for all diseases with one or more corresponding gene, as opposed to restricting to the diseases with at least 20 genes. As expected, the inclusion of these diseases with fewer genes are known lowers the prediction performance, yet it remains significantly higher than the random expectation. Given that the drug disease proximity is not biased with respect to number of disease genes, the drop in the AUC can be attributed to the diseases with less genes being genetically less understood. On the other hand, as several diseases used in the original analysis are broader categories involving more specific conditions, we assess the effect of excluding the broader MeSH disease categories from the analysis (e.g., liver cirrhosis is removed and liver cirrhosis biliary is kept). To do this we identify the disease pairs that have substantial portion of their genes in common (i.e. that have a Jaccard index higher than 0.5) and keep only the specific MeSH term in the MeSH hierarchy (lower in the hierarchy). We observe that the resulting prediction accuracy is comparable to the AUC using all the diseases.

In the original analysis, we assume that the known drug targets are typically the therapeutic targets (for which the drug is intended for). To check whether the analysis depends on the number of targets a drug has, we limit the analysis to those drugs that had at least three targets. In line with our expectation, the AUC does not change substantially compared to using all drugs. Similarly, to confirm that proximity can pick drug-disease associations for drugs whose targets are not disease genes, we repeat the analysis excluding

the drug-disease pairs in which all drug targets are also disease genes ($d_c = 0$). The AUC values are only slightly lower, suggesting that relative proximity can successfully identify indirect relationships between drugs and diseases.

Supplementary References

[1] Assaf Gottlieb, Gideon Y Stein, Eytan Ruppin, and Roded Sharan. PREDICT: a method for inferring novel drug indications with application to personalized medicine. *Mol Syst Biol*, 7:496, June 2011.

[2] Feixiong Cheng, Chuang Liu, Jing Jiang, Weiqiang Lu, Weihua Li, Guixia Liu, Weixing Zhou, Jin Huang, and Yun Tang. Prediction of drug-target interactions and drug repositioning via network-based inference. *PLoS Comput Biol*, 8(5):e1002503, May 2012.

[3] Simone Daminelli, V. Joachim Haupt, Matthias Reimann, and Michael Schroeder. Drug repositioning through incomplete bi-cliques in an integrated drug–target–disease network. *Integr. Biol.*, 4(7):778–788, June 2012.

[4] Joachim von Eichborn, Manuela S. Murgueitio, Mathias Dunkel, Soeren Koerner, Philip E. Bourne, and Robert Preissner. PROMISCUOUS: a database for network-based drug-repositioning. *Nucl. Acids Res.*, 39(suppl 1):D1060–D1066, January 2011.

[5] Michael Kuhn, Monica Campillos, Ivica Letunic, Lars Juhl Jensen, and Peer Bork. A side effect resource to capture phenotypic effects of drugs. *Mol. Syst. Biol.*, 6:343, 2010.

[6] Guanghui Hu and Pankaj Agarwal. Human disease-drug network based on genomic expression profiles. *PLoS ONE*, 4(8):e6536, August 2009.

[7] Marina Sirota, Joel T. Dudley, Jeewon Kim, Annie P. Chiang, Alex A. Morgan, Alejandro Sweet-Cordero, Julien Sage, and Atul J. Butte. Discovery and preclinical validation of drug indications using compendia of public gene expression data. *Sci Transl Med*, 3(96):96ra77, August 2011.

[8] Clare Pacini, Francesco Iorio, Emanuel Gonalves, Murat Iskar, Thomas Klabunde, Peer Bork, and Julio Saez-Rodriguez. DvD: An R/Cytoscape pipeline for drug repurposing using public repositories of gene expression data. *Bioinformatics*, 29(1):132–134, January 2013.

[9] Justin Lamb, Emily D. Crawford, David Peck, Joshua W. Modell, Irene C. Blat, Matthew J. Wrobel, Jim Lerner, Jean-Philippe Brunet, Aravind Subramanian, Kenneth N. Ross, Michael Reich, Haley Hieronymus, Guo Wei, Scott A. Armstrong, Stephen J. Haggarty, Paul A. Clemons, Ru Wei, Steven A. Carr, Eric S. Lander, and Todd R. Golub. The Connectivity Map: Using Gene-Expression Signatures to Connect Small Molecules, Genes, and Disease. *Science*, 313(5795):1929–1935, September 2006.

[10] Francesco Iorio, Roberta Bosotti, Emanuela Scacheri, Vincenzo Belcastro, Pratibha Mithbaokar, Rosa Ferriero, Loredana Murino, Roberto Tagliaferri, Nicola Brunetti-Pierri, Antonella Isacchi, and Diego di Bernardo. Discovery of drug mode of action and drug repositioning from transcriptional responses. *Proc. Natl. Acad. Sci. U.S.A.*, 107(33):14621–14626, August 2010.

[11] Tobias Sing, Oliver Sander, Niko Beerenwinkel, and Thomas Lengauer. ROCR: Visualizing classifier performance in r. *Bioinformatics*, 21(20):3940–3941, October 2005.

[12] Thomas Rolland, Murat Taan, Benoit Charloteaux, Samuel J. Pevzner, Quan Zhong, Nidhi Sahni, Song Yi, Irma Lemmens, Celia Fontanillo, Roberto Mosca, Atanas Kamburov, Susan D. Ghiassian, Xinping Yang, Lila Ghamsari, Dawit Balcha, Bridget E. Begg, Pascal Braun, Marc Brehme, Martin P. Broly, Anne-Ruxandra Carvunis, Dan Convery-Zupan, Roser Corominas, Jasmin Coulombe-Huntington, Elizabeth Dann, Matija Dreze, Amlie Dricot, Changyu Fan, Eric Franzosa, Fana Gebreab, Bryan J. Gutierrez, Madeleine F. Hardy, Mike Jin, Shuli Kang, Ruth Kiros, Guan Ning Lin, Katja Luck, Andrew MacWilliams, Jrg Menche, Ryan R. Murray, Alexandre Palagi, Matthew M. Poulin, Xavier Rambout, John Rasla, Patrick Reichert, Viviana Romero, Elien Ruyssinck, Julie M. Sahalie, Annemarie Scholz, Akash A. Shah, Amitabh Sharma, Yun Shen, Kerstin Spirohn, Stanley Tam, Alexander O. Tejeda, Shelly A. Trigg, Jean-Claude Twizere, Kerwin Vega, Jennifer Walsh, Michael E. Cusick, Yu Xia, Albert-László Barabási, Lilia M. Iakoucheva, Patrick Aloy, Javier De LasRivas, Jan Tavernier, Michael A. Calderwood, David E. Hill, Tong Hao, Frederick P. Roth, and Marc Vidal. A proteome-scale map of the human interactome network. *Cell*, 159(5):1212–1226, November 2014.

[13] Jing-Dong J. Han, Denis Dupuy, Nicolas Bertin, Michael E. Cusick, and Marc Vidal. Effect of sampling on topology predictions of protein-protein interaction networks. *Nat Biotech*, 23(7):839–844, July 2005.

[14] Andrea Franceschini, Damian Szklarczyk, Sune Frankild, Michael Kuhn, Milan Simonovic, Alexander Roth, Jianyi Lin, Pablo Minguez, Peer Bork, Christian von Mering, and Lars J. Jensen. STRING v9.1: protein-protein interaction networks, with increased coverage and integration. *Nucl. Acids Res.*, 41(D1):D808–D815, January 2013.

[15] Jordi Mestres, Elisabet Gregori-Puigjan, Sergi Valverde, and Ricard V. Sol. Data completenessthe achilles heel of drug-target networks. *Nat Biotech*, 26(9):983–984, September 2008.

[16] Michael Kuhn, Damian Szklarczyk, Andrea Franceschini, Christian von Mering, Lars Juhl Jensen, and Peer Bork. STITCH 3: zooming in on proteinchemical interactions. *Nucl. Acids Res.*, 40(D1):D876–D880, January 2012.

[17] Steven H. Brown, Peter L. Elkin, S. Trent Rosenbloom, Casey Husser, Brent A. Bauer, Michael J. Lincoln, John Carter, Mark Erlbaum, and Mark S. Tuttle. VA national drug file reference terminology: a cross-institutional content coverage study. *Stud Health Technol Inform*, 107(Pt 1):477–481, 2004.

[18] Minoru Kanehisa, Susumu Goto, Masahiro Hattori, Kiyoko F. Aoki-Kinoshita, Masumi Itoh, Shuichi Kawashima, Toshiaki Katayama, Michihiro Araki, and Mika Hirakawa. From genomics to chemical genomics: new developments in KEGG. *Nucl. Acids Res.*, 34(suppl 1):D354–D357, January 2006.

[19] Maureen A. Sartor, Alex Ade, Zach Wright, David States, Gilbert S. Omenn, Brian Athey, and Alla Karnovsky. Metab2mesh: annotating compounds with medical sub-

ject headings. *Bioinformatics*, 28(10):1408–1410, May 2012.
